# Physics-Inspired Frequency-Decoupled Network for Remote Sensing Image Dehazing

**DOI:** 10.3390/s26103124

**Published:** 2026-05-15

**Authors:** Hao Yang, Xiaohan Chen, Gang Xu

**Affiliations:** 1 School of Mechanical Engineering, Hubei Engineering University, Xiaogan 432000, China; hyang027@163.com; 2 National Engineering Research Center of Fiber Optic Sensing Technology and Networks, Wuhan University of Technology, Wuhan 430060, China; 345110@whut.edu.cn

**Keywords:** remote sensing image dehazing, frequency decoupling, physical guidance, learnable lifting wavelet, state space model, spectral fidelity

## Abstract

**Highlights:**

**What are the main findings?**

- A correlation is identified between the estimated haze density and the optimal discretization step size of Mamba, which supports the adaptive modulation of the state space model’s receptive field for remote sensing image dehazing.
- Luminance-aware constraints can help reduce spectral distortion and over-dehazing artifacts typically encountered in high-reflectance ground objects.

**What are the implications of the main findings?**

- The physics-inspired frequency-decoupling framework helps alleviate spectral–spatial coupling interference and improves the interpretability of state space models in remote sensing restoration.
- The proposed approach achieves high-fidelity haze removal with favorable spectral and structural consistency, supporting the reliability of downstream remote sensing applications.

**Abstract:**

Remote sensing (RS) imagery often suffers from non-uniform atmospheric scattering, resulting in severe contrast degradation, detail blurring, and spectral distortion. While recent advanced State Space Models (SSMs) offer efficient long-range modeling, they frequently struggle with spectral–spatial coupling interference and lack explicit physical constraints, leading to over-smoothed textures and color biases in high-reflectance regions. In this paper, we propose PhysWave-SSN, a Physics-Inspired Frequency-Decoupled Network specifically designed for high-fidelity RS image dehazing. The architecture employs a task-adaptive frequency-specific screening strategy to effectively isolate structural details from atmospheric interference. Specifically, we first introduce a Frequency-Aware Selection Gate (FASG) that unifies adaptive channel screening with physical transmission estimation, enabling precise recalibration of frequency components. To bridge the gap between physical scattering principles and state space representation learning, we develop a Physics-Informed SSM (PI-SSM), where the discretization step size of Mamba is dynamically modulated by the estimated haze density. This mechanism allows the model to adaptively adjust its spatial receptive field according to local degradation levels, enhancing physical interpretability. Furthermore, a Luminance-Adaptive Fusion Module (LAFM) is presented to protect high-reflectance land covers and maintain spectral consistency. Extensive experiments on multiple RS datasets demonstrate that PhysWave-SSN achieves superior performance, notably attaining a maximum PSNR gain of 2.49 dB while ensuring high structural and spectral fidelity.

## 1. Introduction

Remote sensing imagery captured by satellites or unmanned aerial vehicles is indispensable for diverse earth observation tasks, such as environmental monitoring, urban planning, agriculture inspection, and disaster assessment [1,2]. However, the imaging process is frequently degraded by atmospheric turbid media, particularly haze and fog. Due to the absorption and scattering of light by atmospheric particles, hazy RS images suffer from severe contrast reduction, geospatial detail loss, and spectral distortion. Such degradation not only limits the visual quality of the images but also severely impairs the performance of downstream computer vision tasks, such as land cover classification and object detection. Consequently, effective image dehazing is a vital prerequisite for reliable RS applications [3].

Early research on image dehazing primarily relied on physical scattering models and hand-crafted priors. Central to these approaches is the classical Atmospheric Scattering Model (ASM) [4], which mathematically describes the formation of a hazy image as:

(1) I(x)=J(x)t(x)+A(1−t(x))

where I(x) represents the observed hazy image, J(x) is the latent haze-free radiance to be recovered, A is the global atmospheric light, and t(x) denotes the medium transmission map. The transmission map is typically expressed as $t(x)=e^{-\beta d(x)}$, where β is the scattering coefficient and d(x) represents the scene depth. Since J(x), t(x), and A are all unknown given only I(x), dehazing is essentially a highly ill-posed inverse problem. To constrain the solution space, various priors were developed. For instance, the Dark Channel Prior (DCP) proposed by He et al. [5] is a landmark contribution, built on the observation that most haze-free patches contain pixels with very low intensity in at least one color channel. Following this, various priors were developed, such as the Color Attenuation Prior (CAP) [6]. Beyond channel-based statistics, Fattal [7] introduced the color-lines model based on the observation that pixel values in a small image patch exhibit a one-dimensional distribution. Furthermore, Berman et al. [8] proposed a non-local dehazing algorithm, which assumes that the colors of a haze-free image can be approximated by a few hundred distinct colors that form clusters in RGB space. Although these prior-based methods are computationally efficient and do not require training data, they often suffer from significant limitations, such as over-saturation in high-reflectance areas (e.g., white rooftops) and inaccurate haze thickness estimation in complex, non-homogeneous atmospheric conditions [9,10].

While Convolutional Neural Networks (CNNs) have advanced image restoration, their bounded receptive fields limit their ability to model large-scale, non-homogeneous atmospheric scattering [11,12,13,14,15]. For instance, representative CNNs like AOD-Net [16] often produce chromatic distortions in dense-haze RS regions due to insufficient global contextual awareness. To address this, Transformer-based architectures [17,18,19] leverage self-attention for global modeling, though they suffer from quadratic computational complexity [20,21]. Furthermore, models like DehazeFormer [18] tend to over-smooth fine-grained textures in complex RS scenes, as their global attention lacks sub-band awareness and physical scattering constraints.

Recently, Mamba-based state space models have emerged as a hardware-efficient alternative, achieving global context modeling with linear complexity [22,23]. While variants like VmambaIR [24], WEamba [25], and the dual-attention network by Sui et al. [26] demonstrate strong dehazing capabilities, they process spatial frequencies uniformly without physics-informed guidance. Consequently, they remain susceptible to spectral–spatial interference and high-reflectance ambiguity specific to RS acquisition geometry.

Beyond the architectural advances described above, it is imperative to address the unique physical challenges that distinguish remote sensing (RS) dehazing from natural image restoration. A primary discrepancy lies in spectral sensitivity; RS sensors are high-precision radiometrically calibrated instruments designed to capture physically interpretable spectral radiance for downstream quantitative analyses rather than mere visual aesthetics. Furthermore, the spatial distribution of haze in RS imagery presents significant complexity due to the vast coverage areas, where topographic variations and meteorological gradients lead to intense non-uniformity that often violates the global haze assumptions used in natural scenes. The distinct imaging geometry further compounds these issues, as the near-nadir, top-down perspective lacks the depth-based perspective cues prevalent in ground-level photography. This absence of depth information makes it inherently challenging to statistically differentiate between high-albedo ground objects, such as white rooftops, and localized thick haze, frequently leading to artifacts in conventional dehazing outputs.

**Figure 1:**
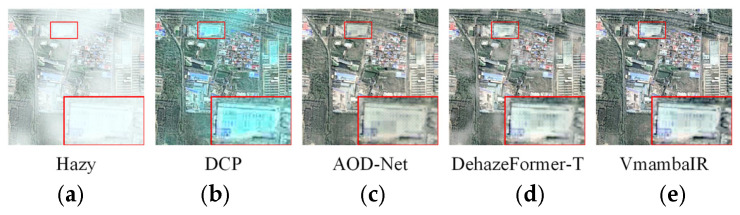
Visual performance degradation and typical failure modes of state-of-the-art dehazing methods on RS imagery. Compared to the hazy input (**a**,**b**) prior-based DCP [5] and (**c**) CNN-based AOD-Net [16] exhibit noticeable over-darkening on high-albedo rooftops. Meanwhile, (**d**) Transformer-based DehazeFormer-T [18] and (**e**) SSM-based VmambaIR [24] leave persistent residual haze, demonstrating an empirical need for our proposed physics-inspired approach. The red box highlights a typical local region, with its magnified view displayed in the lower right corner for intuitive comparison.

These theoretical limitations are supported by our empirical observations across diverse RS datasets. As illustrated in Figure 1, when processing scenes with high-albedo urban structures, existing methods frequently exhibit observable performance degradations: prior-based methods [5] and CNN baseline [16] tend to produce noticeable over-darkening artifacts. Similarly, leading Transformer [18] and SSM-based frameworks [24] often leave persistent residual haze in regions of high optical depth, struggling to adequately adapt to the spatial non-uniformity characteristic of large-scale RS acquisitions. These observations underscore an empirical necessity for a dehazing framework that explicitly incorporates frequency-decoupling and physical guidance.

Against this background, directly applying existing SSM-based frameworks to realistic remote sensing dehazing still faces three challenges. First, they often suffer from irrelevant feature interference due to the lack of an adaptive frequency-selection mechanism. Second, the absence of explicit physical guidance prevents these models from accurately characterizing non-homogeneous haze distributions. Third, high-reflectance ground objects are frequently misidentified as dense haze, leading to severe spectral distortion and unnatural darkening artifacts. These limitations collectively restrict the modeling accuracy and spectral fidelity of current dehazing methods [27,28].

To address the aforementioned issues, we propose PhysWave-SSN, a physics-inspired frequency-decoupled network designed to synchronize atmospheric scattering priors with state-space modeling. The main contributions are summarized as follows:

(1) Frequency-decoupled guidance mechanism: We design a Frequency-Aware Selection Gate (FASG) that utilizes learnable lifting wavelets to disentangle haze-relevant low-frequency components from high-frequency structural noise. This mechanism enables the synchronization of adaptive channel screening with physical transmission estimation, providing spatially consistent guidance to suppress interference while preserving underlying spectral cues.
(2) Physics-inspired discretization in SSM: We introduce a Physics-Inspired State Space Model (PI-SSM), which re-engineers the discretization step size Δ of Mamba by embedding the atmospheric scattering prior as an inductive bias. This structural modification allows the model to dynamically adjust its receptive field according to local haze density, addressing the inherent limitations of generic SSMs in characterizing non-homogeneous degradation.
(3) Luminance-aware spectral preservation: To mitigate the misidentification of high-reflectance land covers, we develop a Luminance-Adaptive Fusion Module (LAFM). By incorporating a luminance-sensitive protection mechanism, the module adaptively constrains the dehazing intensity in bright regions, facilitating a balance between global visibility enhancement and local spectral fidelity for complex land-surface targets.

## 2. Materials and Methods

**Figure 2:**
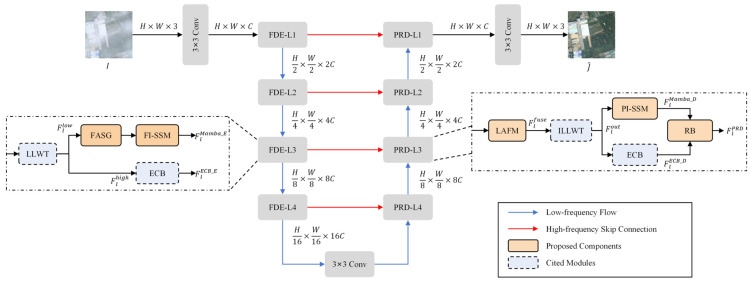
The overall architecture of the proposed PhysWave-SSN. Our model features a frequency-decoupled U-shaped structure. The blue lines denote the low-frequency flow, which conveys global structural information to deeper encoder stages. The red lines represent the high-frequency skip connections, which deliver fine-grained textures and edge details directly to the PRD.

The proposed PhysWave-SSN is structured as a variant of the widely adopted U-Net paradigm, leveraging its symmetric encoder–decoder topology and skip-connection design which have proven effective for multi-scale image restoration. While adhering to this established macro-architecture, the primary innovations of PhysWave-SSN lie in the physics-informed, frequency-decoupled design of its internal modules—specifically the FASG, PI-SSM, and LAFM. These components are integrated within the U-Net framework to address domain-specific challenges such as non-uniform haze and spectral distortion in RS imagery. Concretely, the model employs a hierarchical 4-stage structure with channel dimensions of {C, 2C, 4C, 8C} respectively, where the base channel width is set to C = 32. Within this framework, traditional spatial-domain operations are enhanced by frequency-domain analysis and luminance-aware fusion mechanisms. As illustrated in Figure 2, the framework consists of a Frequency-Decoupled Encoder (FDE) and a Progressive Restoration Decoder (PRD), which are bridged by a bottleneck 3×3 convolution layer. Given a hazy input image $I\in R^{H\times W\times 3}$, the model first extracts shallow features via an initial 3×3 convolution. These features are then sequentially processed by the 4-stage FDE. At each hierarchical stage *l*, a Learnable Lifting Wavelet Transform (LLWT) [29] adaptively decomposes the features into low-frequency components $F_{l}^{low}$ and high-frequency components $F_{l}^{high}$. The $F_{l}^{low}$ branch, capturing the bulk of atmospheric scattering, is processed sequentially by the FASG and the PI-SSM to generate the latent feature $F_{l}^{Mamba\_E}$. Simultaneously, the $F_{l}^{high}$ branch leverages an edge-enhanced convolutional block (ECB) to produce the skip-connection signal $F_{l}^{ECB\_E}$.

Following the encoder stages, the PRD initiates the feature reintegration process. In each PRD stage, the LAFM integrates the skip feature $F_{l}^{ECB\_E}$ with the upsampled feature $F_{l+1}^{PRD}$ from the deeper stage to produce a fused representation $F_{l}^{fuse}$. This fused signal, along with the feedback from the preceding decoder stage, is fed into the Inverse Learnable Lifting Wavelet Transform (ILLWT) [29] to reconstruct the intermediate spatial feature $F_{l}^{out}$. To further enhance visual fidelity, $F_{l}^{out}$ is branched into a parallel dual-path structure: an SS2D Mamba branch establishes global contextual consistency ($F_{l}^{Mamba\_D}$) while a parallel ECB branch explicitly sharpens localized geometric edges ($F_{l}^{ECB\_D}$). These two paths are finally integrated and calibrated by the Refinement Block (RB) to yield the refined stage output $F_{l}^{PRD}$.

The proposed PhysWave-SSN follows an asymmetric dual-domain design rationale to balance haze removal and detail reconstruction. Specifically, the FDE is dedicated to frequency-domain analysis and decoupling. By decomposing features into distinct sub-bands, the encoder mitigates the spatial-frequency coupling conflict, allowing the model to isolate atmospheric scattering from complex terrestrial textures in a simplified spectral space. Conversely, the PRD shifts the focus toward spatial-domain synthesis and joint calibration. Upon reverting to the spatial domain via the ILLWT, the decoder leverages a dual-path refinement strategy—employing the PI-SSM for global context consistency and the ECB for local structural sharpening. This transition from frequency-domain ‘purification’ to spatial-domain ‘refinement’ ensures that the network not only effectively suppresses non-homogeneous haze but also achieves superior radiometric accuracy and geometric fidelity in the final restored imagery.

### 2.1. Frequency-Decoupled Encoder

Remote sensing images degraded by non-uniform haze inherently suffer from the spatial-frequency coupling conflict, which severely restricts the performance of conventional dehazing networks. Haze-related degradation primarily corresponds to low-frequency, global, and slowly varying components caused by atmospheric scattering and ambient light. In contrast, ground structures, edges, textures, and fine land-cover details belong to high-frequency, local, and fast-varying components that carry critical spatial information. When traditional spatial-domain models (e.g., CNNs, Transformers, and vanilla Mamba) conduct unified feature learning, they inevitably entangle these two types of components. Global long-range modeling tends to over-smooth high-frequency details, while local texture enhancement fails to capture large-scale non-uniform haze variations. This undesirable entanglement and mutual interference constitute the spatial-frequency coupling conflict.

Formally, given a hazy image feature $F\in R^{H\times W\times C}$, it can be decomposed into frequency components as:

(2) $$F=F_{low}+F_{high}$$

where $F_{low}$ denotes low-frequency haze and illumination components, and $F_{high}$ represents high-frequency structural and textural details. For a generic spatial feature extractor M(·) without frequency decoupling, the feature learning process can be expressed as:

(3) $$M(F)=M(F_{low}+F_{high})$$

Due to the lack of frequency-aware processing mechanisms, such extractors cannot distinguish haze components from structural details, leading to severe attenuation of high-frequency information:

(4) $$M(F)\approx {\tilde{F}}_{low}+\alpha_{h}{\tilde{F}}_{high},\;\alpha_{h}<1$$

where $\alpha_{h}$ denotes the suppression coefficient of high-frequency components. This leads to texture blurring, edge loss, and structural distortion in the dehazed results.

To address this critical issue, we design a Frequency-Decoupled Encoder based on the LLWT, which adaptively decomposes hazy features into low-frequency and high-frequency sub-bands. This divide-and-conquer strategy enables independent modeling of global haze and local structures, thus effectively alleviating the spatial-frequency coupling conflict.

#### 2.1.1. Learnable Lifting Wavelet Transform

**Figure 3:**
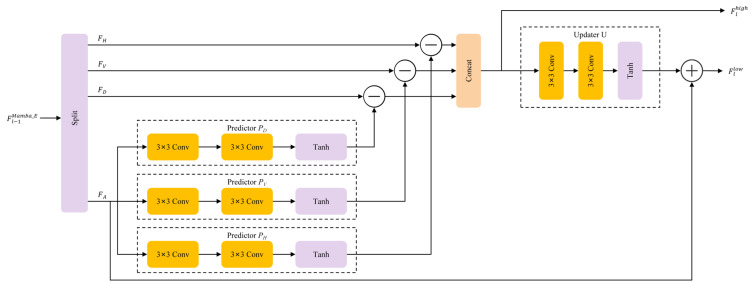
Detailed architecture of the Learnable Lifting Wavelet Transform. The module adaptively decomposes the input feature map into high-frequency ($F_{l}^{high}$) and low-frequency ($F_{l}^{low}$) components through a mathematically reversible pipeline, which consists of spatial splitting, predictive residual extraction via predictors ($P_{H},P_{V},P_{D}$), and adaptive refinement via an updater (U).

To achieve adaptive frequency decoupling while preventing the information loss inherent in conventional downsampling (e.g., strided convolution), we leverage the LLWT as a mathematically robust foundation for frequency decomposition, which provides the necessary sub-bands for our proposed FASG to perform adaptive selection. Unlike traditional wavelets with fixed filters that are suboptimal for the spatially variant haze in RS imagery, the LLWT optimizes the lifting scheme through learnable operators to achieve content-aware decomposition. The detailed architecture is illustrated in Figure 3.

Given an input feature map $F_{l-1}^{Mamba\_E}\in R^{H\times W\times C}$, the LLWT partitions it according to spatial positions into four sub-sequences: $F_{A}$ (even-row, even-column), $F_{H}$ (even-row, odd-column), $F_{V}$ (odd-row, even-column), and $F_{D}$ (odd-row, odd-column). Each sub-sequence retains C channels while reducing the spatial resolution to half. Following the splitting, the frequency decomposition is performed via two adaptive steps:

- Predictive Residual Extraction: Using the approximation component $F_{A}$ as the reference, the predictor P(·) estimates the detail residuals in horizontal, vertical, and diagonal directions. This generates the high-frequency components $F_{l}^{high}$:
  (5) $$F_{l}^{high}=Cat(F_{H}-P_{H}(F_{A});F_{V}-P_{V}(F_{A});F_{D}-P_{D}(F_{A}))$$
- Adaptive Update: The extracted high-frequency residuals are then utilized by the updater U(·) to refine $F_{A}$, yielding the enhanced low-frequency approximation $F_{l}^{low}$
  (6) $$F_{l}^{low}=F_{A}+U(F_{l}^{high})$$

Here, $P_{H}$, $P_{V}$, $P_{D}$ and U are learnable operators, each implemented as two 3×3 convolutional layers followed by a *Tanh* activation function. This configuration allows the network to adaptively separate low-frequency atmospheric light from high-frequency structural textures based on the degree of local degradation.

To ensure training stability and optimal gradient flow in the early stages, we adopt an identity-equivalent initialization strategy by initializing the internal convolutional weights in P and U to zero, ensuring that P(·)≈0 and U(·)≈0. In this state, the LLWT effectively reduces to a lossless spatial rearrangement (similar to Pixel-unshuffle), preserving the complete information flow:

(7) $$F_{l}^{low}\approx F_{A},\;F_{l}^{high}\approx Cat(F_{H},F_{V},F_{D})$$

As training proceeds, the LLWT evolves into a task-specific frequency decoupler rather than a fixed transform. It effectively decomposes the input into $F_{l}^{low}$, which captures the smooth illumination and global haze distribution, and $F_{l}^{high}$, which encapsulates fine spatial structures and detailed textural variations. This decomposition ensures that the subsequent modeling can be conducted in a frequency-targeted manner, wherein the energy-concentrated $F_{l}^{low}$ provides a stable basis for the long-range dependency modeling in the SS2D Mamba, while the sparse but informative $F_{l}^{high}$ serves as the ideal input for the edge-enhancement operations in the ECB. Combined with the symmetric ILLWT, this design maintains strict information conservation while enabling the fine-grained restoration of frequency-specific details.

#### 2.1.2. Frequency-Aware Selection Gate

**Figure 4:**
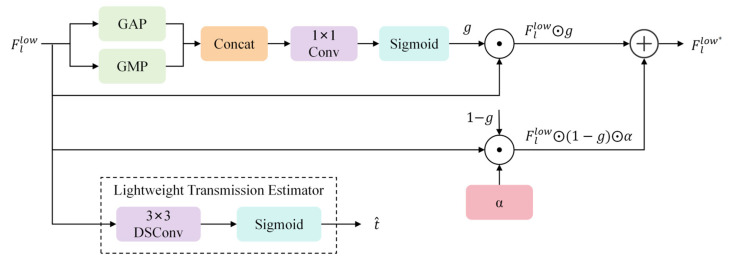
Detailed architecture of the Frequency-Aware Selection Gate.

Although the low-frequency components $F_{l}^{low}\in R^{H\times W\times C}$ generated by the LLWT effectively encapsulate the global haze distribution, they are concurrently intertwined with essential intrinsic illumination and coarse-grained land-cover details. Processing the unfiltered $F_{l}^{low}$ directly via SS2D Mamba may lead to undesirable over-smoothing or spectral distortion. To mitigate these issues and maintain the spectral purity of the background, we propose the FASG. The detailed architecture of the FASG module is illustrated in Figure 4.

The FASG module operates as a lightweight, adaptive filtering mechanism. The process begins by aggregating global context through dual-path pooling. Specifically, we apply Global Average Pooling (GAP) and Global Maximum Pooling (GMP) to $F_{l}^{low}$ along the spatial dimensions to capture both the mean intensity of the haze and the extreme atmospheric light responses:

(8) $$v_{avg}=GAP(F_{l}^{low}),\;v_{max}=GMP(F_{l}^{low})$$

where $v_{avg},v_{max}\in R^{1\times 1\times C}$. These descriptors are concatenated and processed by a 1×1 convolution layer to model inter-channel dependencies, followed by a Sigmoid activation function to generate the frequency selection mask $g\in [0,\;1{]}^{1\times 1\times C}$:

(9) $$g=\sigma ({Conv}_{1\times 1}([v_{avg};v_{max}]))$$

The mask g acts as a gatekeeper; values closer to 1 indicate haze-dominant channels that require intensive modeling by the subsequent Mamba block, while values closer to 0 represent stable background low-frequencies. To avoid potential information loss in non-haze regions, we implement an adaptive residual screening strategy to obtain the refined feature ${F_{l}^{low}}^{*}$:

(10) $${F_{l}^{low}}^{*}=F_{l}^{low}⊙g+F_{l}^{low}⊙(1-g)\cdot \alpha$$

In this formulation, ⊙ denotes element-wise multiplication. The parameter α is a learnable scalar coefficient, initialized at 0.1. This design allows the network to autonomously decide the retention ratio of “stray” low-frequency information. By preserving a fraction of the original signal even in suppressed regions, FASG ensures the structural integrity of the remote sensing scene while effectively isolating haze-related features for the Mamba-based reconstruction.

In addition to channel-wise feature screening, the FASG also functions as a Lightweight Transmission Estimator. Since the low-frequency component $F_{l}^{low}$ inherently encapsulates global haze density and illumination intensity, it serves as a natural prerequisite for physical parameter inference. To ensure spatial consistency in the estimated haze distribution, we employ a 3×3 depthwise separable convolution (DSConv) followed by a Sigmoid activation layer. This structure captures local neighborhood dependencies while maintaining a minimal computational footprint. Specifically, the spatial-wise transmission map $\hat{t}\in [0,\;1{]}^{H\times W\times 1}$ is generated as follows:

(11) $$\hat{t}=\sigma ({DSConv}_{3\times 3}(F_{l}^{low}))$$

where σ denotes the Sigmoid function, which constrains the transmission values within the physically significant range of [0, 1]. This estimated $\hat{t}$ is then integrated into the subsequent PI-SSM block to guide the physics-aware discretization process. For deeper stages, $\hat{t}$ is downsampled via bilinear interpolation to align with the spatial resolution of the corresponding feature maps. This dual-purpose design—generating both the channel mask g and the transmission map $\hat{t}$—endows the model with an effective physical prior with minor computational overhead.

#### 2.1.3. Physics-Informed State Space Modeling

**Figure 5:**
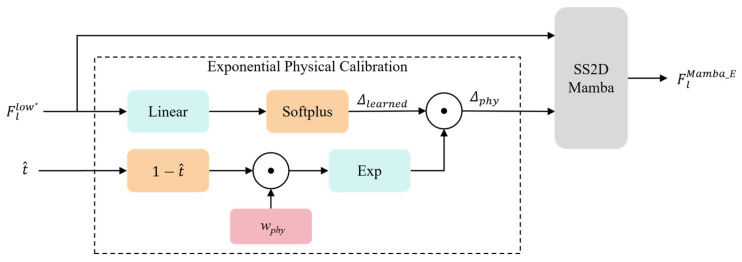
Schematic of the PI-SSM block with the proposed Exponential Physical Calibration (EPC) mechanism, which dynamically modulates the discretization step size based on the exponential decay prior derived from the atmospheric scattering model.

While standard State Space Models, such as SS2D Mamba, excel at long-range dependency modeling, they operate primarily as data-driven black boxes, frequently overlooking the deterministic physical laws governing atmospheric degradation. In remote sensing imagery, haze density is typically non-homogeneous, and the effective range of reliable visual information is strictly dictated by the atmospheric scattering process. To bridge the gap between deterministic physical laws and data-driven sequence modeling, we propose an Exponential Physical Calibration (EPC) strategy. This approach is grounded in the inherent mathematical correspondence between the atmospheric scattering process and the state space discretization mechanism. The schematic of the PI-SSM and the EPC mechanism is depicted in Figure 5.

As established in the classical physical model, the transmission map t(x) dictates the non-linear attenuation of visual information, following an exponential decay relationship with scene depth:

(12) $$t(x)=e^{-\beta d(x)}$$

In dense haze regions, valid local signals diminish rapidly, necessitating the adaptive expansion of the spatial receptive field to aggregate broader contextual information for structural recovery.

Concurrently, the continuous-time SSM is transformed into a discrete-time representation via a discretization step size Δ, where the state transition is governed by:

(13) $$h_{k}=\exp(\Delta A)h_{k-1}+Bu_{k}$$

In SSMs, the state matrix A is typically initialized with negative values to ensure system stability. Consequently, a smaller discretization step Δ causes the decay factor exp(ΔA) to approach unity, thereby preserving the hidden state $h_{k-1}$ over longer sequence distances. This mathematical property dictates that to effectively expand the receptive field in heavily degraded regions, the model must explicitly decrease Δ.

The modulation of the continuous-time discretization step size Δ is inherently sensitive to numerical perturbations. In the standard atmospheric scattering model, the relationship between scene depth $d\left(x\right)$ and transmission $t\left(x\right)$ is defined as Equation (12), yielding $d\left(x\right)\propto -\ln\left(t\left(x\right)\right)$. However, directly embedding the logarithmic mapping into the discretization process introduces severe numerical instability. Specifically, in regions with dense haze where t →0, the unbounded nature of $\lim_{t\to 0}\left[-ln\left(t\right)\right]=+\infty$ would trigger gradient explosion and state divergence during the recurrent formulation of SSM.

To ensure mathematical robustness, PI-SSM employs a bounded exponential proxy function, expressed as:

(14) $$\phi (x)=\exp(-w_{phy}\cdot (1-\hat{t}))$$

Here, $\hat{t}\in [0,\;1]$ is the estimated spatial transmission, and $\left(1-\hat{t}\right)$ serves as a numerically stable surrogate for atmospheric haze density. $\omega_{phy}$ is a learnable channel-wise scaling parameter initialized with a small positive value. This bounded proxy guarantees numerical stability and uniqueness across extreme non-homogeneous regions:

(1) Dense Haze Regions (t →0): The modulation factor asymptotically approaches a strictly positive lower bound $\exp\left(-w_{phy}\right)$. This avoids singularity while enforcing a locally constrained receptive field to capture dense structural degradation.
(2) Clear or High-Reflectance Regions (t →1): The modulation factor converges to $\exp\left(0\right)=1$. Consequently, the modulated step size $\Delta^{\prime }$ smoothly degenerates to the standard Mamba step size Δ($\Delta^{\prime }\to \Delta$). This boundary condition ensures that the network preserves original contextual information without over-modulating clear or highly reflective structures (e.g., clouds or metallic roofs).

Therefore, rather than enforcing a rigid physical constraint, the proposed proxy serves as a numerically stable inductive bias. It aligns the discretization step size of Mamba with the physical degradation trend while maintaining the topological stability of the state space equations.

Consequently, the physics-modulated discretization step $\Delta_{phy}$ is formulated as the element-wise product of the data-driven baseline and the physical modulator:

(15) $$\Delta_{phy}=\Delta_{learned}⊙\phi (x)=\Delta_{learned}⊙\exp(-w_{phy}⊙(1-\hat{t}))$$

(16) $$\Delta_{learned}=Softplus(Linear({F_{l}^{low}}^{*}))$$

where $\Delta_{learned}$ provides the fundamental data-driven spatial representation, and ⊙ denotes element-wise multiplication. By embedding this bounded inverse process directly into the state transition matrix, the PI-SSM evolves from a generic sequence processor into a robust, physics-grounded inference engine.

The proposed physics-inspired multiplicative design offers critical theoretical and practical advantages. Primarily, it perfectly aligns the sequence modeling mechanism with atmospheric physics: in dense haze regions ($\hat{t}\to 0$), the modulator ϕ(x) exponentially decays, proactively shrinking $\Delta_{phy}$. This reduction forces the Mamba block to retain historical states longer, effectively expanding the spatial aggregation window to capture the global structural dependencies essential for inferring obscured details. Conversely, in clear or high-reflectance areas ($\hat{t}\to 1$), the modulation term ϕ(x) seamlessly converges to unity, allowing $\Delta_{phy}$ to revert to the data-driven baseline $\Delta_{learned}$, thereby preserving high-frequency details and preventing over-dehazing artifacts. Furthermore, by utilizing a bounded exponential proxy instead of a strict logarithmic inverse, the EPC inherently guarantees numerical robustness, effectively circumventing the unstable gradients that typically compromise physics-informed network training.

Subsequently, the SS2D Mamba is performed using the physics-aware parameters $\Delta_{phy}$. By grounding the state space transitions in the atmospheric scattering model, PI-SSM transforms the generic sequence modeling of Mamba into a physics-aware inference engine. This enables the network to maintain superior spectral fidelity and structural consistency, particularly in complex remote sensing scenes with varying haze thickness.

#### 2.1.4. Edge-Enhanced Convolutional Block

**Figure 6:**
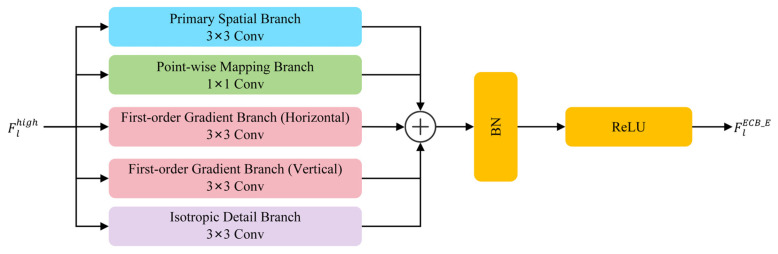
Detailed architecture of the Edge-Enhanced Convolutional Block.

To complement the global modeling of the Mamba block, which can inadvertently smooth out local edge details due to its long-range perception mechanism, the high-frequency path employs an ECB [30]. The ECB is specifically designed to process the $F_{l}^{high}$ component, which encapsulates crucial spatial gradient information such as geometric contours and fine textures of ground objects. Through a structural re-parameterization architecture, as illustrated in Figure 6, the ECB explicitly compensates for structural information that might be lost during the global dehazing process. While the ECB has been proven to effectively capture localized gradients in low-level vision tasks, its integration within our PRD facilitates a synergistic dual-path refinement strategy. By operating in parallel with the PI-SSM, the ECB in our framework performs a targeted structural sharpening that was not explored in original edge-enhancement contexts, thereby ensuring superior geometric fidelity in the final dehazed results.

During the training phase, the ECB leverages a multi-branch parallel topology to comprehensively perceive structural transients from different orders and directions, comprising four distinct components:

(1) Primary Spatial Branch: utilizes a standard 3×3 convolution to capture foundational local semantic features.
(2) Point-wise Mapping Branch: employs a 1×1 convolution for cross-channel linear projection, preserving the original high-frequency responses.
(3) First-order Gradient Branch: incorporates horizontal ($G_{x}$) and vertical ($G_{y}$) Sobel operators to explicitly extract directional edge responses, anchoring prominent anisotropic boundaries such as building facades and road networks.
(4) Isotropic Detail Branch: utilizes a Laplacian operator to capture second-order, direction-independent high-frequency signals, which are highly sensitive to isolated point-like textures and intricate vegetation details.

To explicitly enforce the physical priors of gradient perception from the very beginning of the training process, the initialization strategy for these branches is highly specific. The convolutional kernels within the First-order Gradient Branch and the Isotropic Detail Branch are deterministically initialized with the discrete predefined matrices of the standard Sobel and Laplacian operators, respectively. These prior-constrained spatial templates are applied depth-wise across all input channels, paired with learnable scaling factors to adaptively adjust the gradient response intensity. In contrast, the 3×3 and 1×1 convolutions in the Primary Spatial and Point-wise Mapping branches are initialized using standard random distribution techniques to ensure general semantic mapping capability without structural bias.

Mathematically, the intermediate feature maps from these parallel branches are aggregated via element-wise summation, followed by Batch Normalization (BN) and a Rectified Linear Unit (ReLU) activation function. The output of the ECB, denoted as $F_{l}^{ECB\_E}$, is formulated as:

(17) $$F_{l}^{ECB\_E}=\delta (BN(P_{S}(F_{l}^{high})+P_{M}(F_{l}^{high})+G_{x}(F_{l}^{high})+G_{y}(F_{l}^{high})+L(F_{l}^{high})))$$

where δ(·) represents the ReLU activation function; $P_{S}$ and $P_{M}$ denote the primary spatial branch with a 3×3 convolution and the point-wise mapping branch with a 1×1 convolution, respectively; $G_{x}$ and $G_{y}$ signify the first-order gradient branches employing horizontal and vertical Sobel kernels for directional edge perception; and L characterizes the isotropic detail branch utilizing a Laplacian kernel for omnidirectional texture enhancement.

Crucially, while this multi-branch design enhances the representational capacity during training, it incurs no additional computational overhead during deployment. Through the principle of structural re-parameterization [31], the linear operators within the five distinct paths are mathematically collapsed into a single, unified 3×3 convolutional kernel for inference.

From the physical perspective of RS monitoring tasks, this dual-order gradient-aware design holds substantial practical value. Because RS images typically possess extremely high spatial frequencies, critical topologies are highly susceptible to damage during conventional dehazing procedures. By integrating multi-order gradient guidance and seamless inference acceleration, the ECB ensures that the images retain accurate geometric topologies after the removal of atmospheric scattering effects, thereby providing high-quality foundational support for downstream tasks such as land cover classification and object detection.

### 2.2. Progressive Restoration Decoder

#### 2.2.1. Luminance-Adaptive Fusion Module

**Figure 7:**
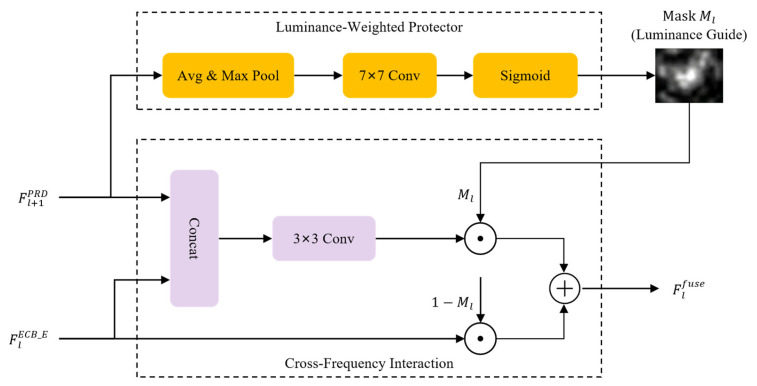
The LAFM and its internal LWP mechanism.

The primary objective of the PRD is to reconstruct haze-free RS scenes with high fidelity and spectral consistency by leveraging multi-scale frequency features from the encoder. However, a significant challenge in RS image restoration is the spectral distortion that occurs when high-reflectance land covers—such as white rooftops, concrete pavements, or turbid water bodies—are misinterpreted as dense haze patterns. To mitigate this issue, we introduce the LAFM within the skip connections of the U-shaped architecture. The internal structure and the information flow of the LAFM are shown in Figure 7.

The core of the LAFM is the Luminance-Weighted Protector, a mechanism designed to move beyond indiscriminate feature injection. Instead of treating all regions equally, the LWP generates a spatial guidance map $M_{l}$ by aggregating the channel-wise average and maximum responses of the features. Physically, average pooling effectively captures the statistical distribution of the global haze field, while max pooling sensitively identifies local salient luminance cues corresponding to highly reflective ground objects. The mathematical formulation of this process is as follows:

(18) $$M_{l}=\sigma \left(W_{p}\left(\left[AvgPool(F_{l-1}^{PRD});MaxPool(F_{l-1}^{PRD})\right]\right)\right)$$

where [·;·] denotes channel-wise concatenation, $W_{p}$ represents a 7×7 convolutional layer used to compress the concatenated representation into a single-channel density mask, and σ is the sigmoid activation function. This mask functions as a robust gating signal that adaptively identifies and shields sensitive regions susceptible to over-dehazing.

Subsequently, the Cross-Frequency Interaction (CFI) gate utilizes this mask to dynamically modulate the integration of multi-band information. The fusion logic is defined as:

(19) $$F_{l}^{fuse}=\phi [F_{l-1}^{PRD};F_{l-1}^{ECB\_E}]⊙M_{l}+F_{l-1}^{ECB\_E}⊙{(1-M}_{l})$$

where ϕ denotes a 3×3 convolutional feature alignment operator that extracts structural anchors from the concatenated frequency inputs, and ⊙ represents element-wise multiplication.

Through this self-guided gating mechanism, the LAFM facilitates adaptive feature recovery by modulating the information flow according to local luminance intensities. To this end, it injects high-frequency details into heavily degraded regions where $M_{l}\approx 1$, and simultaneously suppresses excessive structural modifications in inherently clear or high-brightness areas where $M_{l}\approx 0$. By adaptively modulating the feature flow, the LAFM enhances spectral fidelity through physical-level constraints, thereby reducing potential color biases and artificial artifacts commonly found in conventional spatial-domain fusion methods. This improved spectral integrity provides a more reliable foundation for subsequent quantitative RS analyses, such as biophysical parameter estimation and land cover classification.

#### 2.2.2. Inverse Learnable Lifting Wavelet Transform

**Figure 8:**
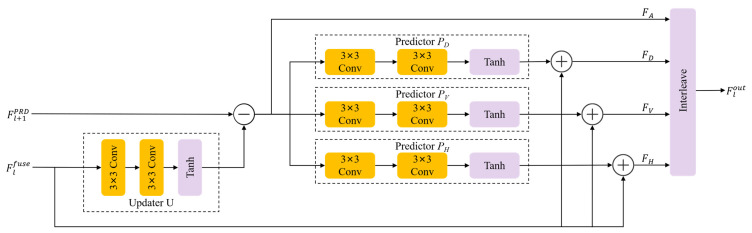
Schematic of the ILLWT.

To reconstruct the spatial resolution of the dehazed features while maintaining information conservation, we design the ILLWT as the functional counterpart to the LLWT in the decoding stages. In RS restoration, traditional upsampling methods like bilinear interpolation or transposed convolution often suffer from checkerboard artifacts or the loss of fine-grained geometric structures. In contrast, the ILLWT performs a mathematically lossless frequency recombination, transforming the decoupled sub-bands back into a single feature map with doubled spatial resolution, the detailed pipeline of which is illustrated in Figure 8.

The ILLWT is implemented as a symmetric inverse lifting scheme, which systematically reverses the adaptive prediction and update operators formulated in Section 2.1.1. Given the enhanced low-frequency features $F_{l-1}^{PRD}$ and the modulated high-frequency features $F_{l}^{fuse}$ from the LAFM, the reconstruction process is executed through the following steps:

Inverse Update: The first step retrieves the approximation component $F_{A}$ by subtracting the update response from the low-frequency input:

(20) $$F_{A}=F_{l-1}^{PRD}-U(F_{l}^{fuse})$$

Inverse Prediction: Subsequently, the original spatial sequences $F_{n}$ are recovered by adding the predictive responses back to the high-frequency residuals:

(21) $$F_{n}=F_{l}^{fuse}+P_{n}(F_{A}),\;n\in \{H,V,D\}$$

Once the four sub-sequences $\left\{F_{A},F_{H},F_{V},F_{D}\right\}$ are reconstructed, an Interleave operation is performed to merge these components into a single high-resolution feature map $F_{l}^{out}$. Because P and U share the same learnable weights as their counterparts in the encoder, this inverse transformation ensures that the structural anchors and spectral information extracted during the decomposition phase are accurately re-aligned in the spatial domain.

The reconstructed spatial representation $F_{l}^{out}$ subsequently serves as the shared input for the dual-path spatial refinement stage. Specifically, it is simultaneously propagated into the SS2D Mamba and ECB branches of the PRD to generate the global-context-aware feature $F_{l}^{Mamba\_D}$ and the edge-enhanced feature $F_{l}^{ECB\_D}$, respectively (as illustrated in Figure 2).

#### 2.2.3. Refinement Block

To further eliminate residual artifacts and calibrate the spatial-spectral consistency after the frequency-to-spatial reconstruction, we introduce a refinement block at the final stage of the PRD. Unlike the previous layers that focus on multi-scale reconstruction, the RB is designed to perform fine-grained adjustment by dynamically reconciling global contextual cues with local structural details.

**Figure 9:**
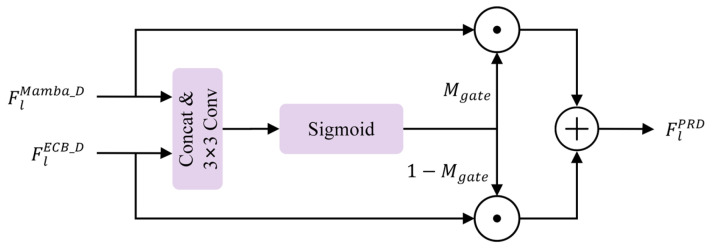
Architecture of RB with Feature-driven Gated Fusion.

As illustrated in Figure 9, the RB serves as the terminal integration unit of the PRD, processing the outputs from the parallel SS2D Mamba and ECB branches. To achieve an optimal fusion of these distinct representations, we propose a Feature-driven Gated Fusion mechanism. Specifically, given the global feature $F_{l}^{Mamba\_D}$ and the local edge feature $F_{l}^{ECB\_D}$, the block first concatenates them to form a joint representation. This joint feature is then passed through a gating network, which consists of a 3×3 convolution followed by a Sigmoid activation function, to generate a spatial-wise fusion weight $M_{gate}\in [0,1]$. The mathematical formulation of the gating weight is as follows:

(22) $$M_{gate}=\sigma (Conv([F_{l}^{Mamba\_D};F_{l}^{ECB\_D}]))$$

The gating weight $M_{gate}$ serves as a dynamic regulatory mechanism that perceives the reliability of each branch at every spatial location. For instance, in regions with homogeneous background haze, the gate prioritizes the global modeling results from the Mamba branch. Conversely, in areas with intricate geometric structures or sharp object boundaries, the gate assigns higher confidence to the ECB branch. The final refined output of the decoder stage, $F_{l}^{PRD}$, is synthesized via a complementary gating operation:

(23) $$F_{l}^{PRD}=M_{gate}⊙F_{l}^{Mamba\_D}+(1-M_{gate})⊙F_{l}^{ECB\_D}$$

By adopting this feature-driven gating strategy, the RB avoids the potential risk of feature over-smoothing that might arise from static weighting. Instead, it ensures that the restored image maintains both long-range spectral uniformity and sharp local contrast, which are vital for subsequent RS interpretation tasks.

### 2.3. Optimization Objectives

To facilitate stable convergence and ensure that the restored images adhere to both structural fidelity and physical consistency, we formulate a comprehensive optimization objective. The total loss function $L_{total}$ is defined as a weighted combination of spatial reconstruction, frequency-domain consistency, and physics-guided regularization, expressed as:

(24) $$L_{total}=L_{pix}+\lambda_{fft}L_{fft}+\lambda_{phy}L_{phy}$$

where $\lambda_{fft}$ and $\lambda_{phy}$ are the trade-off hyperparameters that balance the influence of the spectral and physical constraints relative to the spatial reconstruction.

#### 2.3.1. Spatial Reconstruction Loss

For spatial domain supervision, we employ the Charbonnier loss $L_{pix}$ to ensure structural accuracy. Compared with the standard $L_{2}$ loss, the Charbonnier loss provides superior robustness against outliers and better preserves sharp edges in remote sensing textures. It is defined as:

(25) $$L_{pix}=\sqrt{\|J-\hat{J}{\|}^{2}+\eta^{2}}$$

where J and $\hat{J}$ denote the ground truth and the dehazed image, respectively, and η is a penalty constant for numerical stability.

#### 2.3.2. Spectral Fidelity Loss

To mitigate the spectral distortion frequently observed in high-reflectance regions of remote sensing imagery, a frequency-domain consistency loss $L_{fft}$ is introduced. By minimizing the distance in the Fourier space, the network is encouraged to recover high-frequency textures and maintain global color balance. The spectral loss is formulated as:

(26) $$L_{fft}=\|F(J)-F(\hat{J}){\|}_{1}$$

where F(·) denotes the Fast Fourier Transform (FFT) operation. This supervision ensures that the PhysWave-SSN preserves both low-level structural details and high-level spectral characteristics across different frequency bands.

#### 2.3.3. Reliability-Aware Physics-Consistent Regularization

To prevent the dehazing network from converging toward physically implausible solutions, we introduce a physics-consistent regularization term. While existing methods often rely on the DCP [5] as a pseudo-label for transmission estimation, the DCP assumption frequently collapses in remote sensing scenes containing high-reflectance objects, such as white rooftops, concrete pavements, and saline land. To mitigate the risk of “pseudo-label contamination” and ensure spectral fidelity in these challenging regions, we propose a reliability-aware hybrid physical anchor.

Instead of a singular dependence on DCP, we integrate the CAP [6] to provide a more robust estimation of haze density in bright regions. The hybrid physical anchor $t_{hp}(x)$ is formulated as a fusion of the refined DCP-based transmission $t_{dcp}$ and the CAP-derived transmission $t_{cap}$:

(27) $$t_{hp}(x)=\alpha_{d}\cdot t_{dcp}(x)+(1-\alpha_{d})\cdot t_{cap}(x)$$

where $\alpha_{d}$ is a dynamic fusion coefficient. The $t_{cap}$ is estimated based on the linear relationship between scene depth and the difference between brightness and saturation: $d(x)\propto \theta_{0}+\theta_{1}v(x)+\theta_{2}s(x)$, where v(x) and s(x) denote the value and saturation components in the HSV color space, respectively.

To further safeguard the network from erroneous supervision in high-reflectance areas, we introduce a pixel-wise reliability weight $W_{rel}(x)$. This weight serves to penalize the physical loss only in regions where the physical priors are statistically valid. The weight is defined as:

(28) $$W_{rel}(x)=1-\exp\left(-\frac{\|I(x)-A_{c}{\|}^{2}}{2\delta^{2}}\right)$$

where I(x) is the input intensity and $A_{c}$ is the global atmospheric light. This formulation ensures that for pixels whose intensities are close to the atmospheric light (indicating either dense haze or high-reflectance ground objects where priors are less reliable), the weight $W_{rel}$ decreases, thereby preventing the gradients of the physical loss from dominating the reconstruction.

The optimized physics-consistent loss $L_{phy}$ is defined as the weighted squared error between the network-predicted transmission map $\hat{t}(x)$ and the hybrid anchor $t_{hp}(x)$:

(29) $$L_{phy}=\sum_{x}W_{rel}(x)\cdot \|\hat{t}(x)-t_{hp}(x){\|}^{2}+\gamma \|\nabla \hat{t}(x){\|}_{1}$$

where the second term is a Total Variation (TV) penalty to enforce piecewise smoothness. By incorporating this reliability-aware mechanism, the PhysWave-SSN effectively balances data-driven features with physical constraints, significantly reducing color distortions and over-saturation artifacts in high-reflectance RS terrains.

The hyperparameter settings for the optimization objective are determined through empirical sensitivity analysis and follow established benchmarks in image restoration. Specifically, for the loss weights in $L_{total}$, we set $\lambda_{fft}=0.1$ and $\lambda_{phy}=0.01$ to ensure that the spectral and physical constraints provide meaningful guidance without destabilizing the primary spatial reconstruction. Within the proposed reliability-aware physics-consistent regularization, the fusion coefficient is set to $\alpha_{d}=0.5$ to equitably balance the contributions of the DCP and CAP sub-anchors, while the variance parameter in the reliability weight is defined as δ=0.25 to effectively mitigate interference from high-reflectance terrestrial objects. The coefficient γ is set to $10^{-4}$ to maintain a proper balance between the hybrid physical anchor and structural smoothness. Regarding the constant parameters, we set $\eta =10^{-3}$ in the Charbonnier loss to ensure numerical stability. During the training phase, these hyperparameters remain fixed to ensure the reproducibility and robustness of the PhysWave-SSN across different remote sensing datasets.

## 3. Results

### 3.1. Datasets and Evaluation Metrics

To comprehensively evaluate the robustness, generalization capability, and practical value of the proposed method, extensive experiments were conducted on multiple synthetic datasets, two real-world datasets, and a downstream task dataset.

#### 3.1.1. Datasets

Our benchmark suite comprises five datasets designed to evaluate PhysWave-SSN across synthetic distributions, real-world degradation, and downstream applications. SateHaze1K [32] is a synthetic dataset generated via the ASM with three density levels (thin, moderate, and thick haze). It contains 960 training and 135 test pairs at 512 × 512 resolution. This controlled environment allows for evaluating the sensitivity of the PI-SSM’s physics-aware adaptation to varying scattering intensities. RS-Haze [18] consists of 51,300 training and 2700 test pairs (512 × 512), simulating non-homogeneous haze through spatially variant ASM parameters. Unlike uniform benchmarks, its heterogeneous distributions provide a suitable testbed for assessing the FASG’s frequency-selection and the LLWT’s spatial adaptability.

To assess generalization beyond ASM-based assumptions, the RICE-I and RICE-II datasets [33] provide 500 and 736 real-world hazy/clear pairs, respectively. RICE-I covers diverse terrains (mountains, urban areas) and notably includes high-reflectance surfaces. These scenes are essential for validating the LAFM’s ability to suppress over-dehazing artifacts where traditional priors often fail. RICE-II further extends this diversity with thin cloud and mixed haze conditions over arid regions. Finally, the DeepGlobe Road Extraction Dataset [34] is used for extrinsic validation. In this setup, haze is artificially synthesized onto clear road images to evaluate the performance of a downstream segmentation task using DeepLabV3+ [35]. This task-level validation quantifies whether the radiometric and structural improvements achieved by PhysWave-SSN translate into measurable gains for practical RS interpretation tasks.

#### 3.1.2. Evaluation Metrics

To evaluate dehazing performance across the SateHaze1K, RS-Haze, RICE-I, and RICE-II benchmarks, we employ a suite of full-reference metrics. PSNR [36] and SSIM [37] measure pixel-level restoration and structural similarity, respectively. To assess perceptual quality, LPIPS [38] is utilized to reflect human visual consistency. Furthermore, to quantitatively validate spectral fidelity, we incorporate the SAM [39] and ERGAS [40]. SAM measures the spectral vector back-projection to assess color distortion, while ERGAS provides a global indicator of the relative dimensionless error in spectral synthesis. This multi-dimensional approach ensures a balanced assessment of reconstruction accuracy, structural integrity, perceptual naturalness, and spectral fidelity.

### 3.2. Implementation Details

#### 3.2.1. Experimental Environment and Configuration

The proposed PhysWave-SSN was implemented using the PyTorch 2.10 deep learning framework. All experiments, including training, testing, and downstream task evaluation, were conducted on a high-performance workstation equipped with an NVIDIA GeForce RTX 4090 GPU (24 GB memory; NVIDIA Corp., Santa Clara, CA, USA) and an Intel Core i9-13900K CPU (Intel Corp., Santa Clara, CA, USA). To ensure consistent performance comparison, the software environment was standardized with CUDA 11.8 and cuDNN 8.7.0 on an Ubuntu 22.04 LTS operating system.

#### 3.2.2. Training Protocol and Hyper-Parameters

For the dehazing stage, the model was trained using the AdamW optimizer with a weight decay of $1\times 10^{-2}$ and momentum parameters set to $\left(\beta_{1},\beta_{2})=(0.9,0.999\right)$. The initial learning rate was set to $2\times 10^{-4}$ and followed a cosine annealing schedule to gradually reduce the rate to $1\times 10^{-6}$ over the training period. The training process lasted for 300 epochs with a batch size of 8. To enhance the model’s generalization, we applied data augmentation techniques during the training phase, including random horizontal/vertical flipping and rotations of 90°, 180°, and 270°. All input images were resized or randomly cropped to a resolution of 256×256 pixels.

For the downstream road extraction task, we utilized a pre-trained DeepLabV3+ model with a ResNet-50 backbone. The model was fine-tuned on the DeepGlobe dataset after the dehazing preprocessing. The learning rate for fine-tuning was maintained at $1\times 10^{-4}$ to ensure stable convergence of the segmentation head.

#### 3.2.3. Comparison Methods

The performance of the proposed PhysWave-SSN is evaluated against seven state-of-the-art dehazing methods: DCP [5], AOD-Net [16], DehazeFormer-T [18], VmambaIR [24], UCL-Dehaze [41], EENet [42], and CoA [43]. These methods encompass a range of architectural paradigms, including traditional prior-based models, convolutional neural networks (CNNs), and recent Transformer-based and SSM-based frameworks.

To ensure consistency in the comparative analysis, all baseline models were independently retrained from scratch on the RS-Haze and RICE-I datasets using their respective official source codes. Given that dehazing performance can be sensitive to training protocols and preprocessing steps, all methods—including the proposed model—were trained and evaluated under a unified experimental environment (as specified in Section 3.2.1). We strictly followed the hyperparameter configurations and training schedules recommended in the original publications. Furthermore, an identical data augmentation pipeline and consistent data splits were applied across all experiments. This approach minimizes performance variations potentially arising from differing preprocessing or optimization settings, ensuring that the comparative results reflect the intrinsic capabilities of each architectural design.

### 3.3. Quantitative Evaluation

PhysWave-SSN was compared against seven state-of-the-art methods on SateHaze1K and RS-Haze, using metrics for reconstruction accuracy, perceptual quality, and spectral fidelity. As summarized in Table 1, our model achieves the highest PSNR and SSIM across all tested haze densities. While VmambaIR and EENet show competitive results in specific LPIPS and SAM instances, PhysWave-SSN maintains a consistent lead in the majority of metrics. Particularly on the non-homogeneous RS-Haze dataset, it demonstrates a clear margin in both PSNR and spectral consistency (SAM and ERGAS). These results indicate that the integration of frequency-decoupling (LLWT) and physics-inspired modeling (PI-SSM) effectively preserves structural and spectral details under varying atmospheric conditions.

Table 2 presents quantitative results on the real-world RICE-I and RICE-II datasets. Unlike synthetic benchmarks, these datasets contain naturally occurring haze, which tests the model’s generalization to non-uniform atmospheric distributions. On the RICE-I dataset, PhysWave-SSN achieves the highest scores across all metrics, including PSNR (32.85 dB) and the spectral metrics (SAM and ERGAS). On the more complex RICE-II dataset, our method continues to lead in most categories, such as PSNR (32.18 dB), SSIM (0.9182), and ERGAS (12.83). While CoA shows a marginally lower SAM value on RICE-II, PhysWave-SSN demonstrates consistent performance in perceptual consistency and structural reconstruction. These improvements in spectral fidelity and reconstruction accuracy suggest that the model effectively generalizes to natural haze. This also indicates that the LAFM helps prevent high-reflectance land covers from being misidentified as haze, ensuring the preservation of essential ground details.

To bridge quantitative metrics and visual quality, we define their perceptual implications in RS dehazing. The 1.1–2.5 dB PSNR improvement achieved by PhysWave-SSN is consistent with a noticeable reduction in haze veils and the restoration of natural scene luminance, largely attributable to the PI-SSM’s physics-aware constraints. The concurrent SSIM gain (e.g., 0.89 to 0.93 on RICE-I) reflects improved structural coherence, such as sharper building contours and road boundaries, facilitated by the synergistic action of the LLWT and ECB. Furthermore, the reduction in LPIPS indicates enhanced suppression of artifacts and more naturalistic surface textures, which is linked to the FASG’s adaptive sub-band modulation. This framework provides orthogonal evidence of improvements across radiometric accuracy, structural fidelity, and perceptual naturalness.

### 3.4. Qualitative Evaluation

**Figure 10:**
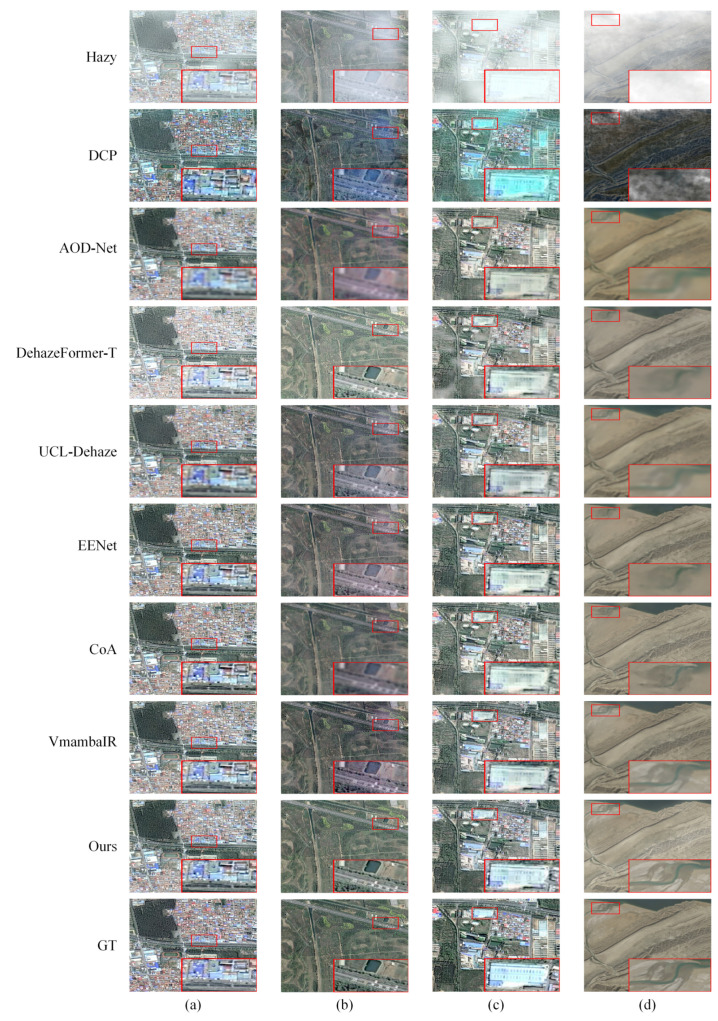
Qualitative visual comparison of dehazing results on the synthetic SateHaze1K and RS-Haze datasets. Columns (**a**–**c**) correspond to the thin, moderate, and thick haze scenarios from the SateHaze1K dataset, while column (**d**) represents a non-homogeneous haze scenario from the RS-Haze dataset. The red box highlights a typical local region, with its magnified view displayed in the lower right corner for intuitive comparison.

To complement the quantitative analysis, we provide a qualitative visual comparison across both synthetic and real-world benchmarks. Figure 10 illustrates the dehazing performance on the SateHaze1K and RS-Haze datasets across varying haze densities (a–d). The results reveal a distinct progression in restoration quality across the evaluated methodologies. Traditional and early CNN-based methods, specifically DCP and AOD-Net, fail to penetrate dense haze, leaving significant residue and noticeable color shifts. While the Transformer-based DehazeFormer-T improves overall visibility, it tends to over-smooth high-frequency textures. More recent benchmarks, including UCL-Dehaze, EENet, and CoA, demonstrate stronger restoration capabilities but still struggle to preserve fine-grained structural integrity in complex areas. Notably, although VmambaIR delivers the most competitive performance among the baselines, it occasionally produces blurred boundaries in intricate regions. In contrast, PhysWave-SSN reconstructs the sharpest edges and most intricate topologies without introducing undesirable artifacts, a visual outcome that reflects the synergy between the frequency-aware decoupling of the LLWT and the geometric guidance of the ECB.

**Figure 11:**
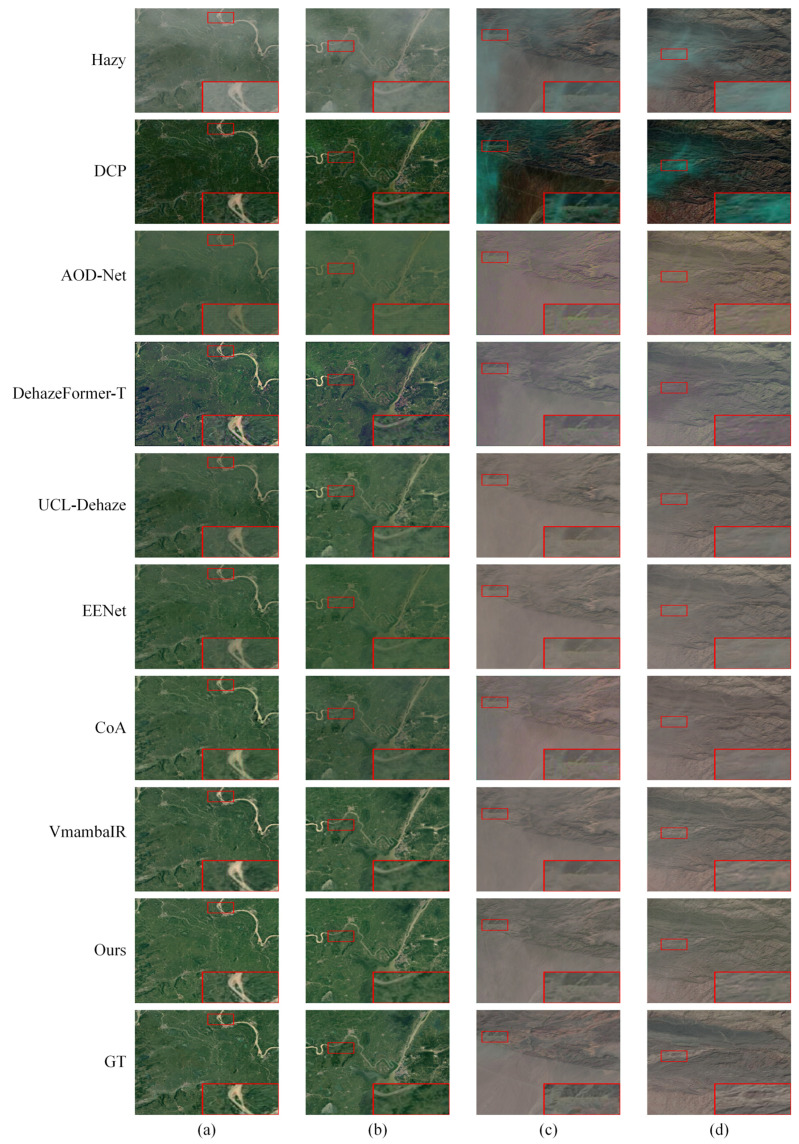
Qualitative visual comparison of dehazing results on the real-world RICE-I and RICE-II datasets. Columns (**a**,**b**) depict scenarios from the RICE-I dataset, and columns (**c**,**d**) present scenarios from the more challenging RICE-II dataset. The red box highlights a typical local region, with its magnified view displayed in the lower right corner for intuitive comparison.

Figure 11 further demonstrates the performance on the real-world RICE-I (rows a–b, vegetated mountains) and RICE-II (rows c–d, arid terrains) datasets. These scenarios highlight the models’ generalization under diverse spectral conditions and non-uniform natural haze. A similar performance gradient is evident, whereby DCP and AOD-Net suffer from severe residual haze and chromatic distortion. Although DehazeFormer-T and more advanced models (UCL-Dehaze, EENet, CoA, and VmambaIR) achieve progressively clearer results, they still exhibit various structural limitations such as edge blurring along ridges or minor texture loss on complex slopes. Conversely, PhysWave-SSN maintains superior color naturalness and structural sharpness across both green and brown terrains. The preservation of such critical topographic details and spectral consistency underscores the practical utility of the LAFM and the ECB/LLWT framework in maintaining fidelity under authentic atmospheric conditions.

### 3.5. Application to Remote Sensing Downstream Tasks

To further validate the practical utility of the proposed PhysWave-SSN, we evaluate its performance on the high-level downstream task of road extraction. Since image dehazing often serves as a prerequisite for subsequent interpretation, the accuracy of semantic segmentation directly reflects the algorithm’s ability to preserve topological structures and spectral information. In this experiment, we treat image dehazing as a foundational preprocessing step for the subsequent segmentation task. We synthesize non-homogeneous haze of varying intensities onto the DeepGlobe Road Extraction Dataset and then utilize a pre-trained DeepLabV3+ model to generate road masks from the dehazed results of various SOTA methods. To provide a rigorous assessment, we adopt Intersection over Union (IoU) and F1-score as quantitative metrics.

Table 3 presents the quantitative road extraction performance under thin and thick haze conditions. Under thin haze, PhysWave-SSN achieves an IoU of 0.5612 and an F1-score of 0.7189, showing a noticeable margin over the second-best method VmambaIR (IoU: 0.5176, F1-score: 0.6821). Prior-based methods such as DCP yield markedly lower scores (IoU: 0.0451), while intermediate methods including EENet and CoA attain moderate performance. Under thick haze conditions, the differences among methods become more pronounced. PhysWave-SSN continues to perform competitively (IoU: 0.6097, F1-score: 0.7575), whereas DCP and AOD-Net degrade considerably (IoU: 0.0141 and 0.0473, respectively), reflecting the increased difficulty of structural recovery under severe atmospheric scattering.

**Figure 12:**
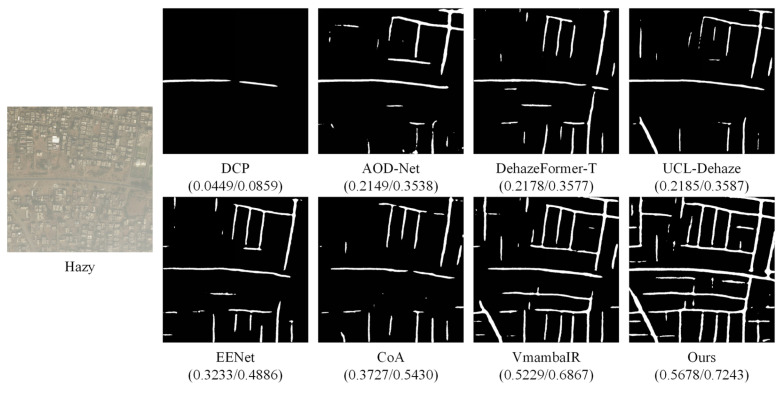
Qualitative comparison of road extraction results using DeepLabV3+ on the DeepGlobe dataset under thin haze conditions. The left panel displays the hazy input image, while the right panel presents the segmented road masks corresponding to eight different dehazing algorithms. The quantitative metrics (IoU/F1-score) are provided below each mask.

**Figure 13:**
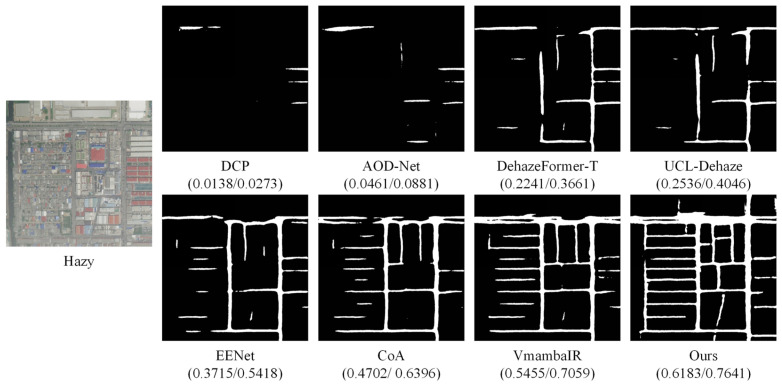
Qualitative comparison of road extraction results using DeepLabV3+ on the DeepGlobe dataset under thick haze conditions. The left panel displays the hazy input image, while the right panel presents the segmented road masks corresponding to eight different dehazing algorithms. The quantitative metrics (IoU/F1-score) are provided below each mask.

Figure 12 and Figure 13 illustrate the visual comparison of road extraction under thin haze and thick haze conditions, respectively. In the thin haze scenario (Figure 12), while most methods can recover the main road skeletons, traditional priors and early CNN-based approaches (e.g., DCP and AOD-Net) suffer from fragmented masks due to residual haze or over-enhancement artifacts. In contrast, PhysWave-SSN produces a more continuous and complete road network. This superiority is attributed to the ECB, which effectively recaptures high-frequency spatial gradients, enabling the segmentation network to identify fine road boundaries and small-scale intersections.

The advantage of PhysWave-SSN is even more pronounced in the thick haze scenario (Figure 13). As the atmospheric scattering increases, ground textures become severely obscured, causing most benchmark algorithms to fail entirely, as evidenced by the significant loss of connectivity and large-scale missing detections in their output masks. However, our model maintains robust performance, extracting a coherent road topology even under extreme degradation. This robustness stems from the LLWT, which decouples global haze modeling from local detail restoration, preventing the SS2D Mamba from over-smoothing structural transients.

### 3.6. Ablation Study

To verify the individual contribution and the necessity of each core component in PhysWave-SSN, we conducted a systematic ablation study on the synthetic RS-Haze and real-world RICE-I datasets. Our analysis is structured into two complementary parts: an incremental integration analysis (Table 4) to evaluate the performance gains of each added module, and a substitution analysis (Table 5) to justify our specific design choices against standard alternatives.

#### 3.6.1. Incremental Analysis

For the incremental study, we established a Baseline (M1) consisting of an SS2D Mamba-based U-Net architecture, which performs feature extraction purely in the spatial domain.

(1) Effectiveness of Frequency-Decoupled Modeling: The integration of LLWT, ILLWT, and ECB forms a frequency-decoupled backbone (M2) for joint spectral decomposition and spatial reconstruction. As indicated in Table 4, this configuration improved the PSNR from 28.29 dB to 29.10 dB and reduced spectral distortion (SAM decreased from 3.055 to 2.862) on the RS-Haze dataset. By adaptively decomposing features into sub-bands and recalibrating structural transients, this strategy allows the network to model atmospheric scattering and ground textures in separate frequency domains, thereby reducing interference between different feature types.
(2) Impact of FASG: The incorporation of the FASG (M3) is observed to further enhance both structural fidelity and spectral consistency. This module increased the SSIM from 0.8522 to 0.8928 and concurrently lowered the SAM to 2.645 and ERGAS to 14.35 on the RS-Haze dataset. By employing frequency-aware sub-band screening, the FASG distinguishes haze-related low-frequency components from land-cover details. This mechanism appears to mitigate the over-smoothing of urban and vegetation structures, a phenomenon often observed in frameworks lacking adaptive frequency selection.
(3) Contribution of PI-SSM: The PI-SSM is evaluated by incorporating the physics-inspired mechanism into the SSM recurrent process (M4). This integration resulted in a substantial performance gain, with the PSNR rising to 33.88 dB and the ERGAS decreasing to 13.71 on the RS-Haze dataset. These improvements suggest that while the standard SS2D Mamba captures long-range dependencies, the PI-SSM assists in rectifying intensity shifts and spectral drifting. By embedding a physical scattering prior via the adaptive discretization parameter $\Delta_{phy}$, the PI-SSM constrains latent feature evolution to align more closely with atmospheric degradation laws.
(4) Necessity of LAFM: As the final integration unit, the LAFM (M5) completes the PhysWave-SSN architecture. The module’s ability to perceive luminance distribution effectively addresses the issue of over-saturation in high-reflectance regions. By guiding the fusion of multi-path features through a learned luminance matrix, the LAFM achieved the most favorable spectral results (SAM: 2.268; ERGAS: 12.54). These results indicate that the luminance-adaptive constraints are instrumental in ensuring that vegetation and urban structures retain their radiometric properties, leading to optimal dehazing performance across both datasets.

#### 3.6.2. Substitution Analysis

To further isolate the contributions and validate the necessity of our specific designs over generic alternatives, we conducted a substitution ablation study as summarized in Table 5. This analysis focuses on whether simpler, standard components could achieve comparable results.

(1) Learnable vs. Fixed Frequency Decoupling: In Variant-A, substituting the learnable LLWT/ILLWT with fixed Haar wavelets led to a degradation in spectral fidelity, with the SAM increasing to 2.481 on the RS-Haze dataset and 3.725 on the RICE-I dataset. These results suggest that while fixed filters can decompose frequencies, they lack the adaptivity required to handle spatially varying haze distributions in remote sensing scenes. The learnable filters in PhysWave-SSN appear more effective at suppressing frequency-aliasing artifacts during reconstruction.
(2) FASG vs. Standard Attention: Replacing the FASG with a standard SE block [44] (Variant-B) resulted in a notable reduction in PSNR (32.13 dB on RS-Haze) and the highest spectral error among the variants (SAM: 2.653, ERGAS: 14.44). While the SE block re-calibrates channel-wise features, it does not explicitly account for frequency-specific haze interference. The performance gap indicates that the FASG’s ability to selectively screen sub-bands based on transmission clues is beneficial for maintaining structural and spectral consistency.
(3) PI-SSM vs. Standard SSM: As demonstrated in Variant-C, replacing the PI-SSM with a standard SS2D Mamba block resulted in a 1.76 dB drop in PSNR and a marked increase in spectral distortion (SAM rose from 2.268 to 2.635). This comparison indicates that although the standard Mamba architecture provides a robust baseline for long-range dependency modeling, it may struggle with the radiometric intensity shifts inherent in hazy remote sensing data. The integration of physical inductive bias ($\Delta_{phy}$) appears to function as a domain-specific constraint that assists the model in rectifying these shifts more accurately than a purely data-driven approach.
(4) LAFM vs. Simple Fusion: The results for Variant-D show that employing simple concatenation and convolution for feature fusion is less effective at preserving high-reflectance regions. Without the luminance-adaptive guidance, the model exhibited increased spectral error (SAM: 2.584 on RS-Haze). The LAFM contributes to the final refinement by ensuring that land-cover details in bright areas are preserved without reaching saturation, thereby maintaining higher radiometric fidelity in the dehazed outputs.

### 3.7. Computational Complexity and Efficiency Analysis

To address the computational efficiency and clarify the source of performance improvements, Table 6 presents a transparent comparison of model parameters (Params) and computational complexity (GMACs) across the evaluated algorithms. The results indicate that the proposed PhysWave-SSN maintains a competitive computational footprint relative to recent Transformer-based and SSM-based baselines. The introduction of the physics-inspired state space modeling and frequency-decoupled mechanisms does not lead to an excessive increase in model scale. This efficiency is facilitated by the structural re-parameterization within the edge-enhanced convolutional block during inference and the lightweight gating design of the frequency-aware selection gate. Consequently, the quantitative metrics and visual improvements observed in the preceding evaluations can be primarily attributed to the effective integration of atmospheric physical priors and adaptive frequency decoupling, rather than relying on a straightforward expansion of parameters and auxiliary modules. This balance between spatial-spectral restoration capability and computational cost supports the practical applicability of the framework in remote sensing interpretation tasks.

## 4. Discussion

### 4.1. Analysis of the Physics-Inspired Frequency-Decoupled Mechanism

The superior performance of PhysWave-SSN, particularly in maintaining spectral fidelity in high-reflectance regions, validates our core hypothesis: purely data-driven SSMs are often insufficient for characterizing the complex optical degradation in non-homogeneous RS scenes. Conventional spatial-domain methods frequently suffer from the “over-correction” trap, where bright land covers—such as concrete pavements and white rooftops—are misidentified as dense haze due to their similar high-pixel-intensity characteristics.

By decoupling features into specific frequency bands via LLWT, our framework effectively isolates atmospheric interference from structural textures. The integration of PI-SSM further bridges the gap between deterministic physical laws and stochastic feature learning. By modulating the discretization step size Δ based on estimated haze density, the model adaptively scales its receptive field, mimicking the variable attenuation of light in the atmosphere. Moreover, the LAFM functions as a critical spectral safeguard; by adaptively governing the feature fusion flow based on luminance priors, it circumvents the common pitfall of color distortion and artificial darkening. This physics-inspired synergy not only enhances visual clarity but also ensures the reliability of restored spectral information, which is fundamental for downstream quantitative remote sensing applications.

### 4.2. Limitations and Failure Analysis

**Figure 14:**
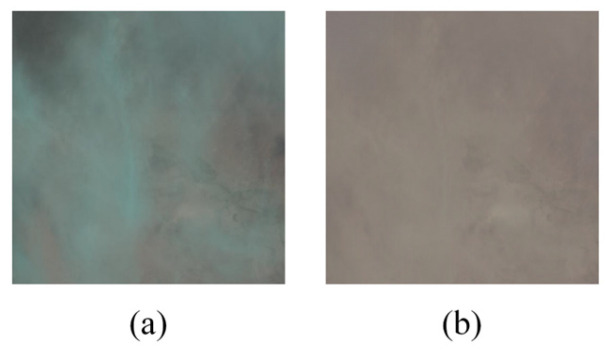
Performance degradation in scenarios with severe atmospheric attenuation: (**a**) Hazy input with dense cloud cover from RICE-II; (**b**) Dehazed result with residual artifacts.

Although PhysWave-SSN exhibits competitive performance in most scenarios, several limitations define its operational boundaries. As illustrated in Figure 14, a primary failure mode occurs under conditions of extreme optical thickness, such as the dense cloud cover found in the RICE-II dataset. When the ground-level signal is almost entirely attenuated, the transmittance approaches zero, leading to a critical depletion of structural information in the input imagery. In such instances, the physical guidance within the PI-SSM may saturate, resulting in recovered regions that exhibit generic textures or mottled artifacts rather than precise ground details (Figure 14b).

Furthermore, the model’s reliance on the ASM can lead to spatially inconsistent results under multi-layer scattering or thin cirrus clouds, where the simplified physical assumptions are partially violated. Future work will explore the integration of complex radiative transfer models and the use of multi-modal data (e.g., SAR) to provide supplementary information in cases of optical signal loss.

## 5. Conclusions

In this paper, we propose PhysWave-SSN, a novel Physics-Inspired Frequency-Decoupled Network designed to address the challenges of spatial-frequency coupling and lack of physical interpretability in remote sensing image dehazing. The framework leverages an LLWT to achieve frequency-specific decomposition, significantly mitigating the conflict between global haze removal and local detail restoration. By introducing the PI-SSM, we incorporate atmospheric scattering priors into the state-space transition process, enabling dynamic adjustment of the scanning receptive field according to local degradation levels. Furthermore, the LAFM is incorporated to protect the spectral integrity of high-reflectance ground objects, effectively suppressing common artifacts such as color bias and darkening.

Experimental results on both synthetic benchmarks and real-world hazy scenes demonstrate that PhysWave-SSN outperforms several existing competitive methods, achieving a maximum PSNR gain of 2.49 dB. The restored imagery exhibits superior structural fidelity and spectral consistency, demonstrating the potential of the proposed physical-neural hybrid design. Future work will focus on extending this physics-inspired Mamba architecture to other low-level RS tasks, such as cloud removal and multi-spectral image pansharpening, while exploring the deployment of the model on edge-computing platforms for real-time satellite data processing.

## Figures and Tables

**Table 1:** Quantitative experiments on synthetic datasets.

Dataset	Metrics	DCP [5]	AOD-Net [16]	DehazeFormer-T [18]	UCL-Dehaze [41]	EENet [42]	CoA [43]	VmambaIR [24]	PhysWave-SSN (Ours)
SateHaze1K-Thin Haze	PSNR ↑	15.15	15.39	22.67	20.81	24.68	24.75	25.52	**27.21**
	SSIM ↑	0.6626	0.7442	0.8363	0.8753	0.9026	0.8917	0.9029	**0.9213**
	LPIPS ↓	0.4782	0.4605	0.2823	0.2912	0.2721	0.2762	0.2709	0.2716
	SAM ↓	12.029	3.622	2.761	3.157	3.486	2.541	2.892	**2.398**
	ERGAS ↓	30.19	25.69	15.24	18.76	22.81	24.15	18.47	**13.36**
SateHaze1K-Moderate Haze	PSNR ↑	15.20	15.97	25.62	24.34	25.32	25.81	26.77	**27.92**
	SSIM ↑	0.7450	0.8169	0.8622	0.9132	0.9105	0.9167	0.9130	**0.9332**
	LPIPS ↓	0.4632	0.4489	0.2790	0.2862	0.2632	0.2668	0.2651	**0.2591**
	SAM ↓	14.381	5.389	4.022	4.162	2.745	5.031	3.598	**2.287**
	ERGAS ↓	40.52	34.86	30.92	28.37	19.05	22.41	16.78	**14.61**
SateHaze1K-Thick Haze	PSNR ↑	14.62	14.44	20.80	19.04	21.13	23.06	23.65	**25.93**
	SSIM ↑	0.6238	0.6913	0.7912	0.7945	0.8427	0.8512	0.8722	**0.9029**
	LPIPS ↓	0.4920	0.4721	0.2837	0.3018	0.2802	0.2687	0.2771	**0.2667**
	SAM ↓	15.602	4.936	3.217	4.705	**2.561**	4.159	3.537	2.843
	ERGAS ↓	38.46	28.31	18.20	17.74	25.46	21.12	27.03	**15.32**
RS-Haze	PSNR ↑	19.99	23.10	29.27	29.82	30.38	31.66	32.12	**34.02**
	SSIM ↑	0.7825	0.8102	0.8365	0.8928	0.9022	0.9135	0.9101	**0.9321**
	LPIPS ↓	0.4631	0.3055	0.2818	0.2233	0.2306	0.1971	0.1960	**0.1957**
	SAM ↓	11.835	4.351	2.937	3.142	4.216	2.403	2.857	**2.268**
	ERGAS ↓	28.77	24.16	16.91	16.31	22.47	23.15	15.27	**12.54**

Note: ↑ indicates that a higher value is better, while ↓ indicates that a lower value is better. Bold fonts and underlined values indicate the best and second-best performance, respectively.

**Table 2:** Quantitative experiments on real-world datasets.

Dataset	Metrics	DCP [5]	AOD-Net [16]	DehazeFormer-T [18]	UCL-Dehaze [41]	EENet [42]	CoA [43]	VmambaIR [24]	PhysWave-SSN (Ours)
RICE-I	PSNR ↑	18.92	20.21	27.52	28.70	29.81	30.28	30.36	**32.85**
	SSIM ↑	0.7516	0.7931	0.8262	0.8729	0.8801	0.8902	0.8925	**0.9301**
	LPIPS ↓	0.4833	0.3285	0.2913	0.2331	0.2446	0.2026	0.2053	**0.1985**
	SAM ↓	11.073	7.204	6.019	5.603	7.295	4.168	3.965	**3.425**
	ERGAS ↓	27.04	20.38	16.34	17.41	15.68	15.17	13.92	**12.25**
RICE-II	PSNR ↑	15.02	18.19	25.37	27.85	28.66	28.72	30.01	**32.18**
	SSIM ↑	0.6025	0.6636	0.7329	0.8177	0.8426	0.8623	0.8711	**0.9182**
	LPIPS ↓	0.5027	0.3589	0.3305	0.2620	0.2631	0.2278	0.2135	**0.2061**
	SAM ↓	14.336	12.167	8.927	6.278	7.689	**4.308**	5.034	4.495
	ERGAS ↓	28.26	22.89	20.73	19.34	15.96	16.51	13.52	**12.83**

Note: ↑ indicates that a higher value is better, while ↓ indicates that a lower value is better. Bold fonts and underlined values indicate the best and second-best performance, respectively.

**Table 3:** Quantitative comparison of road extraction performance on the hazy DeepGlobe dataset restored by various methods.

Condition	Metrics	DCP [5]	AOD-Net [16]	DehazeFormer-T [18]	UCL-Dehaze [41]	EENet [42]	CoA [43]	VmambaIR [24]	PhysWave-SSN (Ours)
Thin Haze	IoU	0.0451	0.2163	0.2195	0.2204	0.3187	0.3694	0.5176	0.5612
	F1-Score	0.0863	0.3557	0.3600	0.3612	0.4834	0.5395	0.6821	0.7189
Thick Haze	IoU	0.0141	0.0473	0.2268	0.2558	0.3683	0.4658	0.5412	0.6097
	F1-Score	0.0278	0.0903	0.3697	0.4074	0.5383	0.6356	0.7023	0.7575

**Table 4:** Ablation study of the proposed components through incremental integration.

Model	Baseline	LLWT&ILLWT&ECB	FASG	PI-SSM	LAFM	RS-Haze					RICE-I				
						PSNR ↑	SSIM ↑	LPIPS ↓	SAM ↓	ERGAS ↓	PSNR ↑	SSIM ↑	LPIPS ↓	SAM ↓	ERGAS ↓
M1	√	×	×	×	×	28.29	0.8407	0.2430	3.055	16.42	27.31	0.8110	0.3002	4.481	14.85
M2	√	√	×	×	×	29.10	0.8522	0.2257	2.862	15.57	28.88	0.8392	0.2569	4.156	14.12
M3	√	√	√	×	×	31.92	0.8928	0.2106	2.645	14.35	30.02	0.8821	0.2237	3.911	13.55
M4	√	√	√	√	×	33.88	0.9226	0.1981	2.517	13.71	32.26	0.9285	0.2002	3.753	12.97
M5	√	√	√	√	√	34.02	0.9321	0.1957	2.268	12.54	32.85	0.9301	0.1985	3.425	12.25

Note: ✓ and ✕ indicate the inclusion and exclusion of the corresponding module, respectively; ↑ indicates that a higher value is better, while ↓ indicates that a lower value is better.

**Table 5:** Quantitative evaluation of substitution ablation studies on the RS-Haze and RICE-I datasets.

Model Variant	Replaced Module	Substituted with	RS-Haze					RICE-I				
			PSNR ↑	SSIM ↑	LPIPS ↓	SAM ↓	ERGAS ↓	PSNR ↑	SSIM ↑	LPIPS ↓	SAM ↓	ERGAS ↓
Variant-A	LLWT/ILLWT	Fixed Haar DWT/IDWT	32.68	0.8921	0.2185	2.481	13.68	31.28	0.8960	0.2258	3.725	12.86
Variant-B	FASG	Standard SE Block	32.13	0.8872	0.2133	2.653	14.44	30.87	0.8851	0.2212	3.961	13.52
Variant-C	PI-SSM	Standard SSM	32.26	0.9087	0.2054	2.635	14.31	31.72	0.9089	0.2096	3.915	13.49
Variant-D	LAFM	Concatenation + 3 × 3 Conv	33.35	0.9220	0.1993	2.584	13.92	31.91	0.9242	0.2027	3.840	13.18
Full Model	-	-	34.02	0.9321	0.1957	2.268	12.54	32.85	0.9301	0.1985	3.425	12.25

Note: ↑ indicates that a higher value is better, while ↓ indicates that a lower value is better.

**Table 6:** Comparison of computational complexity (GMACs) and model parameters (Params) across different dehazing methods.

Method	# Params	MACs
DCP [5]	-	-
AOD-Net [16]	0.002 M	0.12 G
DehazeFormer-T [18]	0.686 M	6.65 G
UCL-Dehaze [41]	19.45 M	78.80 G
EENet [42]	5.44 M	49.61 G
CoA [43]	1.69 M	3.48 G
VmambaIR [24]	28.71 M	150.7 G
PhysWave-SSN (Ours)	29.20 M	41.87 G

Note: # denotes the number of parameters.

## Data Availability

Data are contained within the article.
